# A fibre-optic ultrasound sensor of simple fabrication

**DOI:** 10.1121/10.0028202

**Published:** 2024-08-15

**Authors:** Fadwa Shagroun, Richard James Colchester, Erwin Jozef Alles

**Affiliations:** 1 Department of Medical Physics and Biomedical Engineering, University College London, London, United Kingdom; 2 Wellcome/EPSRC Centre for Interventional and Surgical Sciences, University College London, London, United Kingdom fadwa.shagroun.20@ucl.ac.uk, richard.colchester@ucl.ac.uk, e.alles@ucl.ac.uk

## Abstract

The small size, high sensitivity, and immunity to electromagnetic interference of fibre-optic ultrasound sensors make them highly attractive for applications in biomedical imaging and metrology. Typically, such sensors rely on optically resonant structures, such as Fabry–Perot cavities, that require elaborate fabrication techniques. Here, an alternative fibre-optic ultrasound sensor is presented that comprises a simple deformable and reflective structure that was deposited using simple dip-coating. Interrogation with a laser Doppler vibrometer demonstrated how this sensor achieved a sensitivity, signal-to-noise ratio, and noise-equivalent pressure that outperformed piezoelectric hydrophones, whilst offering a highly miniature form factor, turn-key operation, and simple fabrication.

## 1. Introduction

Fibre-optic ultrasound sensors are an attractive alternative to conventional electronic counterparts in biomedical applications due to their small lateral size (Colchester *et al.*, 2019), high sensitivity (Guggenheim *et al.*, 2017), broad bandwidth (Zhang and Beard, 2015), and immunity to electromagnetic interference (Watt *et al.*, 2023). Several types of optically resonant fibre-optic ultrasound detectors have been presented (Wissmeyer *et al.*, 2018), such as Fabry–Perot cavities (Guggenheim *et al.*, 2017; Zhang and Beard, 2015), ring resonators (Westerveld *et al.*, 2021), and fibre-Bragg gratings (Rosenthal *et al.*, 2011). Each of these detector types utilises an optically resonant structure fabricated at or near the tip of the fibre, enabling exquisite sensitivity, broad bandwidth, or both, in a miniature package.

However, such optically resonant sensors are complicated and costly to manufacture, requiring clean-room facilities, advanced silicon processing equipment, or highly accurate coating deposition. Alternative fibre-optic sensors have been presented that do not rely on optical resonance to achieve pressure sensitivity, which are typically easier and more cost-effective to fabricate.

First, reflectance-type optical hydrophones (Aytac-Kipergil *et al.*, 2023; Staudenraus and Eisenmenger, 1993; Wilkens and Koch, 1999) utilise simple flat-cleaved optical fibre faces as a sensing element, onto which optional dielectric mirrors can be deposited to increase sensitivity. Such sensors derive their pressure sensitivity from pressure-modulated changes in the refractive index in the surrounding water, which are interrogated by measuring the reflectance of the fibre-water interface. Especially in the absence of mirrors, such sensors offer trivial fabrication and simple interrogation schemes (requiring just a stable light source and photodiode), but a limited sensitivity that is typically only relevant for high-intensity focussed ultrasound (HIFU) applications.

Second, various systems have been presented where the fibre-optic sensor forms one arm of a homo- or heterodyne interferometer (Koch, 1999; Koch and Jenderka, 2008; Koch *et al.*, 1997). With such systems, incident ultrasound waves modify the optical path length of the optical fibre, of which the end face was prepared with a rigid reflective layer to improve the detection signal-to-noise ratio (SNR). However, the use of a rigid reflector layer offers a limited sensitivity, and as the optical path length is determined by the entire fibre rather than just the sensing element, such sensors are sensitive to variation in ambient conditions (pressure, temperature), which complicates deployment in dynamic environments, such as the pulsatile blood present during intravascular imaging.

In this work, a novel sensor approach is presented where the fibre-optic sensor contains a deformable and reflective structure at the distal end, which is optically non-resonant and interrogated interferometrically at the proximal end using a broadband laser Doppler vibrometer (LDV). This approach measures the velocity of the reflective sensor surface rather than its displacement or shape (as typically detected through measurements of its optical path length). Thus, a fibre-optic ultrasound sensor is obtained that is readily fabricated and exhibits excellent sensitivity. In addition, the heterodyne LDV employed in this work offers absolute velocity measurements, turn-key operation, and requires no user intervention to achieve or maintain sensitivity. Here, the sensor fabrication and its performance are presented, as well as directions for future work.

## 2. Sensor mechanism and fabrication

When an ultrasound wave impinges on a sensor, the pressure difference (relative to ambient pressure) results in a deformation of the sensing element. For optically resonant sensors, a change in resonance condition results, and ultrasound sensitivity is hence achieved by effectively monitoring the optical path length of the sensor element. In contrast, here a fibre-optic sensor is proposed that is sensitive to the *velocity* of the sensor surface, and hence to the acoustic particle velocity at the sensor boundary, rather than the resonance condition of its (pressure-modulated) geometry. For the proposed sensor, changes in ambient conditions still affect the geometry of the sensor, but merely result in additional components in the sensor surface velocity, rather than a decrease in sensor sensitivity. As environmental changes in biomedical settings (e.g., blood pressure, temperature, thermal expansion of the optical fibre) occur at rates substantially lower than 1 kHz, the ultrasound-modulated surface velocity is readily isolated from ambient fluctuations through simple high-pass filtering.

In the proposed sensor, ultrasound sensitivity is achieved by fabricating a deformable structure on the distal tip of a single-mode optical fibre. This structure is then interferometrically interrogated at the proximal end of the fibre using a LDV that detects the pressure-modulated velocity of the distal surface of the structure. The LDV detection fidelity is optimised by adding a thin reflective layer to the outer surface of the sensing element. Whilst the proximally placed LDV does indeed use optical interferometry, its sensitivity is not influenced by the ambient conditions experienced by the distally located sensing element.

**Fig. 1.**
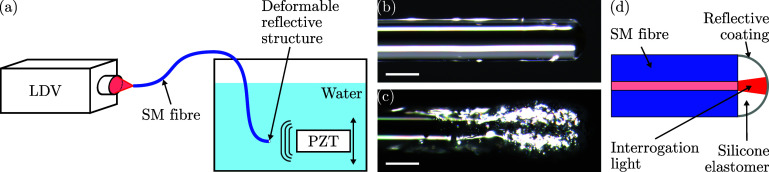
(a) Schematic of the experimental setup, (b) microscope photographs of the non-resonant fibre-optic ultrasound detector before deposition of the reflective layer, (c) microscope photographs of the non-resonant fibre-optic ultrasound detector after deposition of the reflective layer. Scale bars: 100 *μ*m, (d) interrogation light is delivered via a single-mode optical fibre, weakly diverges through a deformable silicone elastomere structure, and is reflected back into the fibre by a concave reflective coating deposited around the elastomer. LDV, laser Doppler vibrometer; SM, single-mode; PZT, piezoelectric transducer.

The proposed sensor design of a deformable, reflective structure deposited on the tip of a fibre allows for the use of simple fabrication techniques. A single-mode optical fibre (SM600, Thorlabs, Bergkirchen, Bavaria, Germany) was stripped down to its cladding, flat-cleaved (CT30, Fujikura, Tokyo, Japan), and subsequently dip-coated with a silicone elastomer (Sylgard 184, Dow, MI), prepared according to manufacturer recommendations) to obtain a deformable “dome-shaped” structure [Fig. 1(b)]. This dome shape has previously been demonstrated to significantly improve ultrasound detection sensitivity and directivity (Guggenheim *et al.*, 2017) by preventing “beam walk-off” where divergent light exiting the fibre is reflected away from the fibre core. To increase the reflectivity of the dome surface, the sensor was dip-coated in a silver paint (Silveriest Silver, Culture Hustle, London, UK) [Fig. 1(c)].

The proximal end of the fibre-optic sensor was placed in the focal point of a broadband LDV [bandwidth: 60 kHz to 24 MHz, focal spot diameter: ca. 15 *μ*m, sensitivity: 1 m/s/V; VibroFlex Neo (VFX-I-110) + Connect (VFX-F-110) + short range lens (VFX-O-SRS), Polytec, Waldbronn, Baden-Württemberg, Germany]. To avoid interference from reflections off the proximal fibre tip, the fibre was angle-cleaved (cleave angle: 8°) and terminated using an Fiber Connector Angled Physical Contact connector. This connector was mounted to a two-dimensional (2D) micropositioner (LM1XY/M + SM1FCA2, Thorlabs, Bergkirchen, Bavaria, Germany) to facilitate accurate alignment.

## 3. Sensor performance

The performance of the sensor was assessed using a commercial piezoelectric ultrasound transducer (centre frequency: 10 MHz, element diameter: 6.35 mm; V312-SU, Evident Corporation, Tokyo, Japan) driven by a pulser (driving amplitude: 400 V; 5077PR, Evident Corporation, Tokyo, Japan). This transducer generated highly consistent and spatially uniform near-planar ultrasound waves, which greatly facilitated transducer alignment and comparison with other sensors. This transducer was mounted on a three-axis translation stage (PT3/M, Thorlabs, Bergkirchen, Bavaria, Germany) and centred in front of the fibre-optic ultrasound sensor at a distance of 1.5 mm [Fig. 1(a)].

To compare the sensor performance, the measurement was repeated using a custom plano–concave fibre-optic Fabry–Perot detector (Zhang and Beard, 2015) (90% sensor reflectivity) and a calibrated piezoelectric needle hydrophone (75 *μ*m diameter; NH0075-SYSTEM, Precision Acoustics, Higher Bockhampton, Dorset UK). Signals from each of these detectors were recorded using a high-speed digitiser (sample rate: 250 MSa/s, bit depth: 16 bits; M4i.4420-x8, Spectrum Instrumentation, Grosshansdorf, Schleswig-Holstein, Germany). A 12 MHz low-pass filter was applied, and no averaging was performed.

**Fig. 2.**
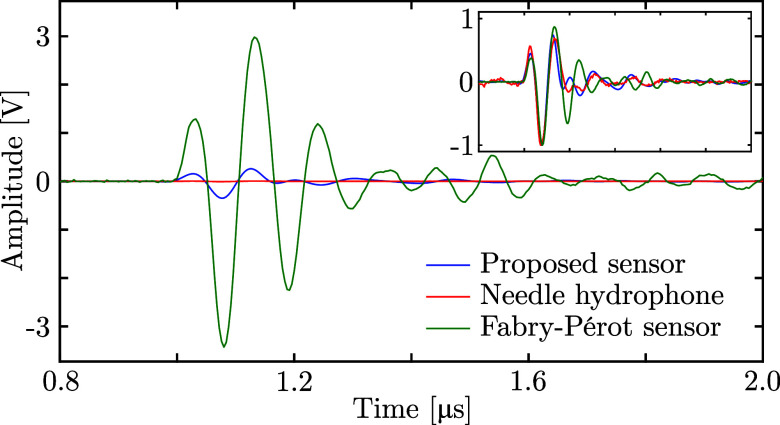
Ultrasound recordings made with three different types of detectors. Relative to the response measured with the Fabry–Perot detector, the amplitudes for the electronic needle hydrophone and proposed non-resonant fibre-optic detector are 10 and 510 times lower, respectively. Inset: signals normalised to –1 to highlight the signal shapes and noise levels.

These measurements (Fig. 2) confirm that the proposed non-resonant fibre-optic ultrasound sensor is highly sensitive, with a sensitivity that is ca. ten times smaller than that of the fibre-optic Fabry–Perot sensor, but ca. 51 times higher than that achieved by the needle hydrophone, despite its significantly smaller lateral size. This resulted in sensor sensitivities of 5100, 510, and 10 mV/MPa for the fibre-optic Fabry–Perot sensor, proposed non-resonant sensor, and needle hydrophone, respectively.

The acoustic field generated by the piezoelectric transducer was observed to exhibit a maximum pressure amplitude of 0.71 ± 0.13 MPa, which was computed using the calibration data (and its uncertainty) supplied for this particular needle hydrophone. In contrast, the LDV yields absolute values for the velocity of the reflective layer, which for a vanishingly thin reflective layer is identical to the ultrasound particle velocity at the water–sensor interface as stipulated by continuity conditions across acoustic interfaces (Cobbold, 2007). Ignoring the mechanical behaviour of the sensing structure, for planar ultrasound waves under normal incidence, such as those considered here, the acoustic pressure *p* and particle velocity *v* are in-phase and related via

p=Zv, (1)

where *Z* is the acoustic impedance of the propagation medium, which for water at room temperature is Z≈1.5 MRayl (Cobbold, 2007). Using Eq. (1), the peak pressure observed with the proposed non-resonant sensor was measured as 0.54 MPa, which is reasonably close to that predicted from the needle hydrophone data. The discrepancy is due to a combination of a slight misalignment between sensor and transducer (resulting in the detection of a non-normal component of the particle velocity), the finite thickness of the reflective layer (where compression of the reflective layer results in apparent reduction in velocity), the curved geometry of the sensor dome (resulting in “spatial averaging” of different components of the velocity field), and the mechanical stiffness of and internal reflections within the sensing element.

For each of the three sensors, the peak SNR was computed as

$$\text{SNR}=10\cdot \;{\text{log}}_{10}\frac{A_{\text{max}}}{\sigma },$$ (2)

where $A_{\text{max}}$ is the peak amplitude of the envelope-detected signal and *σ* is the standard deviation of the first 0.8 *μ*s of the time traces, which did not contain actual ultrasound signals. The SNR values obtained for the Fabry–Perot, proposed non-resonant, and piezoelectric sensors were 31, 21, and 15 dB, respectively. Thus, whilst the fibre-optic Fabry–Perot sensor achieves the highest sensitivity and SNR, the proposed non-resonant sensor significantly outperforms the piezoelectric needle hydrophone in both metrics.

**Fig. 3.**
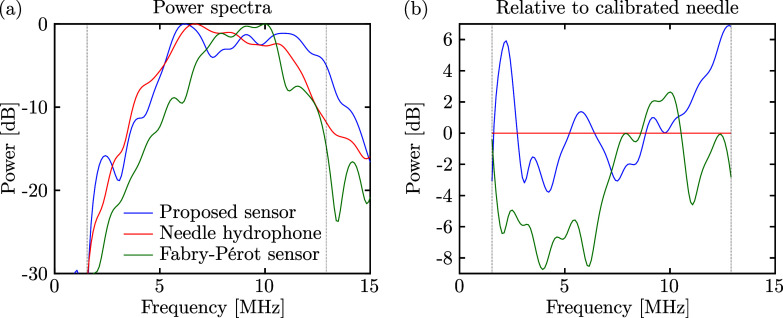
Power spectra of ultrasound recordings made with three different types of detectors, normalised to either (a) 0 dB or (b) to the calibrated needle hydrophone. The dotted vertical lines indicate the SNR-limited bandwidth beyond which the relative power spectra could not reliably be estimated.

The spectral performance of the proposed sensor is presented in Fig. 3, where the power spectra of the three signals of Fig. 2 are shown. The narrow bandwidth of the piezoelectric transducer (ca. 5–12 MHz) as well as the 12 MHz low-pass filter applied to the LDV limit the bandwidth of this study. However, the spectral flatness of the proposed sensor, relative to the signal detected with the calibrated needle hydrophone, is slightly better across the 1.5–13 MHz bandwidth than that observed for the Fabry–Perot sensor.

Furthermore, the noise-equivalent pressure (NEP) was computed for each of the sensors across the 60 kHz to 12 MHz band, using the peak noise amplitudes rather than root mean square values due to the broadband signal being detected (Zhang and Beard, 2015), assuming linear responses for all three sensors. In line with the observations on the sensor SNR, the NEP was lowest for the Fabry–Perot sensor (0.2 kPa), and the proposed sensor (8.3 kPa) achieves a significantly lower NEP compared to the needle hydrophone (47.7 kPa). The sensitivities, SNR, NEP, and dimensions of the sensing elements for the three different sensors are summarised in Table 1. Note that the NEP observed for the Fabry--Perot sensor is higher than that reported in the literature by approximately one order of magnitude (Guggenheim *et al.*, 2017); this is attributed to the use of a sensor exhibiting a lower sensor reflectivity, and hence reduced sensitivity, which was used to prevent saturating the digitiser.

**Table 1.** Summary of the element size, sensitivity, signal-to-noise ratio (SNR), and noise-equivalent pressure (NEP) observed for the various ultrasound sensor types. Sensor dimensions for the fibre-optic sensors are based on the corresponding single-mode fibre mode-field diameters.

	Element size	Sensitivity	SNR	NEP
Sensor type	(*μ*m)	(mV/MPa)	(dB)	(kPa)
Needle	75	10	15	47.7
Proposed	10^a^	510	21	8.3
Fabry–Perot	10^a^	5100	31	0.2

^a^ Estimated assuming Gaussian divergence out of optical fibre.

To demonstrate their potential for temporally extended ultrasound measurements, both the fibre-optic Fabry–Perot and proposed non-resonant sensors were mounted to a motorised stage (MTS50/M-Z8, Thorlabs, Bergkirchen, Bavaria, Germany) and translated across the aperture of the piezoelectric transducer whilst continually recording data. A linear trajectory spanning 20 mm was traversed in 100 *μ*m increments, centred in front of the transducer, and ran parallel to the transducer surface at a distance of 1.5 mm. In each position, 100 recordings were made and averaged, resulting in a line scan acquisition time of ca. 3 min.

**Fig. 4.**
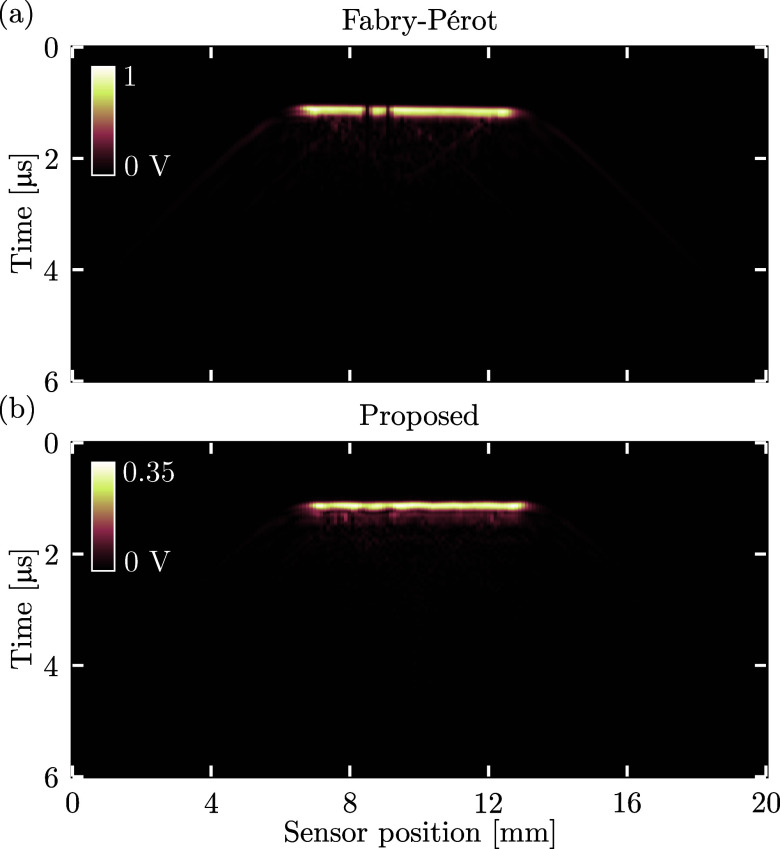
Visualisation of continuous ultrasound recording (“B-scan”) during motorised linear motion across the surface of an ultrasound source, using (a) a fibre-optic Fabry–Perot or (b) proposed non-resonant sensor.

The resulting data (Fig. 4) confirmed that the piezoelectric transducer indeed emitted a near-planar acoustic field, and in addition, that during the 3 min acquisition window the sensitivity of the proposed non-resonant sensor remained constant. In contrast, the Fabry–Perot sensor required continual tracking and adjustment of the interrogation wavelength to ensure optimal sensitivity, which occasionally resulted in signal drop-out corresponding to the darker bands around a sensor position of 9 mm. Both sensors successfully detected the “edge waves” generated by the finite-sized transducer that correspond to the “wing-shaped” events at sensor positions ≤6 and ≥13 mm, confirming that both sensors exhibit sensitivity beyond normal incidence. However, the greater visibility of these edge waves for the Fabry–Perot sensor suggests that the proposed sensor exhibits a more directional sensitivity pattern, despite a similar size of the ultrasound sensing area (cf. Table 1).

## 4. Discussion and conclusion

In this letter, a novel fibre-optic ultrasound sensor is presented that does not derive its sensitivity from optical resonance within the sensor. Ultrasound sensitivity was instead achieved by measuring the velocity of the outer surface of an optically reflective, deformable sensing element by coupling a commercially available, broadband LDV into the proximal end of an optical fibre. Simple dip-coating techniques were employed to fabricate the sensing element, which could, in principle, be performed in the field to fabricate or repair sensors *in situ*. In addition, an insensitivity to the physical dimensions of the proposed sensing structure greatly facilitates mass production—where achieving tight control over the sensor dimensions would require expensive manufacturing techniques—and will hence allow for truly disposable use.

The proposed sensor exhibited a sensitivity, SNR, and NEP significantly exceeding those of a piezoelectric needle hydrophone of similar dimensions. As such, the proposed fibre-optic ultrasound sensor shows great promise for applications in ultrasound metrology. In addition, future alternative sensor designs can be considered that exhibit negligible stiffness or mechanical resonance, for which Eq. (1) is valid. Using, for instance, membrane-style sensing elements (Li *et al.*, 2023) would allow for direct conversion of the absolute velocity measurements obtained from the LDV into quantitative pressure values without the need for sensor-specific calibration.

Whilst a high sensor sensitivity of 510 mV/MPa was observed, the sensor SNR of 21 dB, and thereby the NEP of 8.3 kPa, could be improved further in various ways. First, additional optics could be employed to better match the LDV focal spot size (currently ca. 15 *μ*m) to the mode-field diameter of the single-mode optical fibre (ca. 4.5 *μ*m). This would significantly increase the coupling efficiency into the fibre, as well as its temporal stability, and could be achieved through adding, for instance, graded-index collimators or microscope objectives.

Second, a different reflective compound could be applied that achieves higher optical reflectivity of the sensor at a reduced reflector thickness, such as dielectric mirrors. Whilst this will both improve the sensor SNR and reduce signal distortion due to compression of the reflecting layer, such reflective coatings would likely add to the fabrication complexity and cost of these sensors. Whilst the cost of the optical fibre (ca. £2 per meter) is similar to that of a Fabry–Perot sensor, the simple dip-coating strategy proposed here adds less than £1 to the material cost, whereas dielectric coating runs, such as those required for Fabry–Perot sensors, add thousands per batch.

Third, velocity-controlled dip-coating or alternative deposition methods of the deformable elastomer could be employed to achieve greater control over the curvature of the sensor dome. This will allow accurate tuning of the sensor dome shape to the divergence pattern of the optical fibre, which could further reduce beam walk-off and improve sensor SNR. In addition, this could improve the sensor directivity observed indirectly in Fig. 4, but additional experiments are required to fully characterise and optimise the directional and spectral sensor response as well as its mechanical stability. Whilst in this work the pressure amplitude (0.71 MPa) was limited by the piezoelectric transducer, the LDV employed offers a dynamic range that could in principle detect pressures of up to ca. 9 MPa. Modelling the sensor as a one-dimensional harmonic oscillator operating at a frequency of 10 MHz, the maximum detected surface velocity corresponds to a sensor surface displacement of 4.8 nm, where Hooke's law predicts [for a Young's modulus for polydimethylsiloxane of 1.32 MPa (Moučka *et al.*, 2021)] a surface displacement of 6.6 nm. Whilst the proposed sensor does not exhibit harmonic modes within the experimental bandwidth (cf. Fig. 3)—suggesting broadband sensitivity—the close agreement between these two models confirms that the sensor is operating in the linear regime, and is hence at negligible risk of mechanical damage at the reported pressure level.

The improvements discussed above are expected to increase the performance of the proposed non-resonant fibre-optic ultrasound sensor to approach that observed for state-of-the-art sensors based on Fabry–Perot cavities. Combined with a simple fabrication process, turn-key operation, and the potential for quantitative sensing without the need for calibration measurements, the sensor presented in this work enables reliable, sensitive, and cost-effective sensing for ultrasound and photoacoustic imaging using truly disposable probes, which will facilitate application of ultrasound sensing in highly miniaturised interventional biomedical imaging, in HIFU measurements and metrology, or in non-destructive testing and structural health monitoring.

## Data Availability

The data that support the findings of this study are available from the corresponding author upon reasonable request.
